# PDGF/VEGF signaling controls cell size in *Drosophila*

**DOI:** 10.1186/gb-2009-10-2-r20

**Published:** 2009-02-12

**Authors:** David Sims, Peter Duchek, Buzz Baum

**Affiliations:** 1 Morphogenesis Group, Ludwig Institute for Cancer Research (UCL Branch), Riding House Street, London, W1W 7BS, UK; 2 Current address: The Breakthrough Toby Robins Breast Cancer Research Centre at the Institute of Cancer Research, Chester Beatty Laboratories, Fulham Road, London, SW3 6JB, UK; 3 Current address: Institute of Molecular Biotechnology of the Austrian Academy of Sciences, Dr Bohrgasse, 1030 Vienna, Austria; 4 Current address: MRC Laboratory for Molecular and Cell Biology, University College London, Gower Street, London, WC1E 6BT, UK

## Abstract

**Background:**

In multicellular animals, cell size is controlled by a limited set of conserved intracellular signaling pathways, which when deregulated contribute to tumorigenesis by enabling cells to grow outside their usual niche. To delineate the pathways controlling this process, we screened a genome-scale, image-based *Drosophila *RNA interference dataset for double-stranded RNAs that reduce the average size of adherent S2R+ cells.

**Results:**

Automated analysis of images from this RNA interference screen identified the receptor tyrosine kinase Pvr, Ras pathway components and several novel genes as regulators of cell size. Significantly, Pvr/Ras signaling also affected the size of other *Drosophila *cell lines and of larval hemocytes. A detailed genetic analysis of this growth signaling pathway revealed a role for redundant secreted ligands, Pvf2 and Pvf3, in the establishment of an autocrine growth signaling loop. Downstream of Ras1, growth signaling was found to depend on parallel mitogen-activated protein kinase (MAPK) and phospho-inositide-3-kinase (PI3K) signaling modules, as well as the Tor pathway.

**Conclusions:**

This automated genome-wide screen identifies autocrine Pvf/Pvr signaling, upstream of Ras, MAPK and PI3K, as rate-limiting for the growth of immortalized fly cells in culture. Since, *Pvf2*/*3 *and *Pvr *show mutually exclusive *in vivo *patterns of gene expression, these data suggest that co-expression of this receptor-ligand pair plays a key role in driving cell autonomous growth during the establishment of *Drosophila *cell lines, as has been suggested to occur during tumor development.

## Abstract 2

Pvr and its ligands, Pvf 2 and 3, which are upstream of Ras and PI3kinase, are identified from a genome-wide screen in Drosophila cells, as regulators of cell growth.

## Background

Tissue growth is regulated by a balance of cell growth, proliferation and apoptosis. In many systems, however, cell proliferation and the accumulation of individual cell mass (cell growth) have been shown to be regulated independently, including in mammalian cells [1], fly cells [2] and yeast [3]. This is explained in part by the action of distinct signaling pathways [4]. Ras-Mitogen-activated protein kinase (MAPK) signaling plays the predominant role in the promotion of cell proliferation in mammalian cells, while phospho-inositide-3-kinase (PI3K) and Tor signaling pathways primarily control the ability of individual cells to accumulate mass, through the promotion of ribosome biogenesis and protein translation [5]. To maintain cell size during tissue growth it is therefore important that increases in cell proliferation and the rate of mass accumulation be coordinated. One way to do this is via pathway crosstalk, and there is increasing evidence for direct crosstalk between growth and proliferation signals during normal development [6,7] and in diseased states [7-9]. Historically, screens for genes controlling cell proliferation and growth have been carried out in a tissue context in the animal [10]. Since the discovery of RNA interference (RNAi) [11], however, several groups have taken advantage of the ability to carry out systematic, genome-scale RNAi screens in *Drosophila *cell culture [12-15] to address this problem. Through the design of luciferase and fluorescence activated cell sorting (FACS) based RNAi screens, large numbers of genes have been identified that regulate overall population growth, cell cycle progression, cell size, cell viability and Ras-MAPK signaling [13-15]. In addition, methods have been developed to carry out high content cell-based RNAi screens in *Drosophila *cell culture [16]. Whilst the analysis of such data sets represents a challenge, computational tools have recently been developed that allow an automated analysis of phenotypes from cell images [17]. We have used an automated image analysis pipeline to screen images from a genome-scale, high-content RNAi screen (Sims *et al*., unpublished data) for genes that limit the average size of adherent hemocyte-derived S2R+ cells [16,18]. In this way, we have identified a novel role for autocrine Pvf/Pvr signaling, upstream of both Ras and phospho-inositide-3-kinase (PI3K), as a rate-limiting step in the regulation of *Drosophila *cell size.

## Results

### A genome-scale RNAi screen reveals genes that regulate cell size

**Figure 1:**
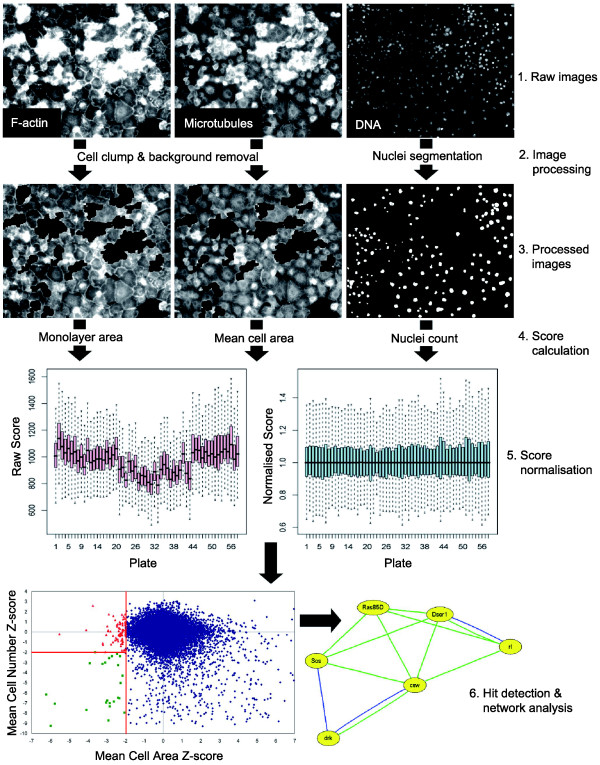
Workflow of the computational analysis of images from a high-content, genome-wide RNAi screen in *Drosophila *cell culture. Raw images of S2R+ cells stained for F-actin, microtubules and DNA were analyzed computationally to calculate the total monolayer area and mean cell area in each image (see Materials and methods for details). Image analysis scores were then normalized across screen plates to create z-scores (see Materials and methods for details). Hits were selected using a z-score threshold of -2 for mean cell area, and a monolayer area z-score of -2 to exclude images with low cell number where small cell size may reflect viability defects (green squares). This, approach yielded 73 putative hits (red triangles), which were examined for known physical and genetic interactions in FLIGHT [20].

To identify regulators of *Drosophila *cell size, a library of approximately 22,000 double-stranded RNAs (dsRNAs) covering 91% of the *Drosophila *genome [14] was screened in 384-well plates. After 5 days of RNAi treatment, S2R+ cells were stained to visualize F-actin, microtubules and DNA and imaged by automated microscopy (Figure 1). The resulting images were computationally analyzed to identify dsRNAs that led to a reduction in average cell area. First, regions of each image containing a monolayer of adherent cells were identified using an algorithm that removes cell clumps and non-cellular background. Individual nuclei within this region were identified, and average cell area calculated by dividing the monolayer area by the nuclear count (Figure 1). Next, scores were normalized using the CellHTS package [19] within the online RNAi database FLIGHT [20] (Figure 1). Results were then filtered to remove dsRNAs that have a profound affect on cell number (Figure 1), which included many housekeeping genes. Finally, manual curation was used to filter out dsRNAs displaying secondary phenotypes, including defects in cell adhesion. The remaining scores were then ranked based on normalized mean cell area, to reveal the 15 dsRNAs that act most potently to reduce S2R+ cell size (Table 1 and Additional data file 1), all of which are conserved between fly and human.

**Table 1:** List of top hits from computational analysis of mean cell area in images from a high-content genome-wide RNAi screen

Fly gene	Human homologue	Z-score	Function
***Sos***	***SOS1***	**-4.09**	**Ras signaling**
***drk***	***GRB2***	**-3.73**	**Ras signaling**
*CG11294*	*CART1*	-3.07	Transcription factor
*gfzf*	*GSTT1*	-3.02	Glutathione transferase
***Pvr***	***PDGF*/*VEGF***	**-2.92**	**Receptor tyrosine kinase**
***rl***	***MAPK1***	**-2.80**	**Ras signaling**
*Sec61alpha*	*SEC61A2*	-2.66	Protein secretion
*Rheb*	*RHEB*	-2.52	Tor signaling
*Nup44A*	*SEH1L*	-2.47	Nuclear pore
***Ras85D***	***KRAS***	**-2.30**	**Ras signaling**
***csw***	***PTPN11 (SHP-2)***	**-2.22**	**Ras signaling**
*CG9306*	*NDUFB9*	-2.20	Mitochondrial electron transport
*CG9300*	*NOL11*	-2.15	Sugar transporter
*fax*	*C6orf168*	-2.04	Axonogenesis
***Dsor1***	***MAP2K1***	**-2.02**	**Ras signaling**

This list was derived from an initial hit list (Additional data file 1) following the confirmation of phenotypes by visual inspection and the exclusion of genes causing other cell shape phenotypes. Genes participating in the Ras signaling pathway are highlighted in bold.

Network analysis of this putative hit list in the database FLIGHT [20] revealed a core set of genes that participate in the Ras/MAPK signaling pathway (*drk*/*Grb2*, *csw*/*SHP-2*, *Sos*, *Ras1*, *Dsor1*/*MEK *and *rl*/*ERK*; Figure 1). In most cases, Ras/MAPK signaling is thought to be activated downstream of ligand binding to a receptor tyrosine kinase [21]. It was notable, therefore, that the screen identified a single receptor tyrosine kinase gene, *Pvr*, that exhibited a strong reduced cell size phenotype (Table 1). Pvr is the sole member of the platelet-derived growth factor (PDGF)/vascular endothelial growth factor (VEGF) family of receptors in *Drosophila*, and has previously been implicated in a range of cellular functions, including migration, proliferation and survival [22-31], but not thus far in the regulation of cell size.

Of the remaining putative hits, only *Rheb*, a component of the growth regulating Tor pathway [32], had a known signaling function. Novel hits were diverse in functions and included *CG9306*, which encodes a component of the electron transport machinery, *Nup44A*, which encodes a nuclear pore component, and several transcription factors. The screen also identified a large number of housekeeping genes, such as ribosomal components *Rps8 *and *Rps18 *and proteosomal components *Prosalpha7 *and *Pomp*, most of which led to a reduction in both cell size and number (Additional data file 1). However, given our focus on cell growth, we limited our further analysis to delineation of the signaling pathway by which *Pvr *and *Ras1 *regulate cell size.

### Validation of hits affecting cell size

**Figure 2:**
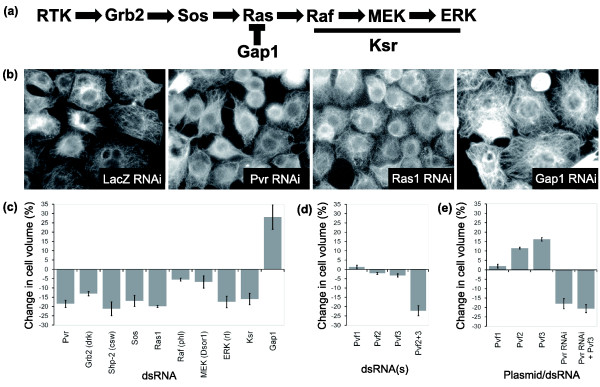
*Pvr*, *Ras1 *and the MAPK pathway control cell size in a *Drosophila *hemocyte-like cell line. **(a) **Schematic of canonical Ras/MAPK signaling. **(b) **Control and RNAi-treated S2R+ cells fixed and stained for microtubules (20× magnification). *Pvr *and *Ras1 *RNAi cause a decrease in cell area, whereas *Gap1 *RNAi causes an increase in cell area compared to control. **(c-e) **Charts of the mean percentage change in volume of RNAi treated or *Pvf *transfected S2R+ cells relative to control cells, as measured using a CASY counter. (c) Silencing of positive regulators of the Ras/MAPK pathway causes a reduction in cell diameter, whereas silencing of *Gap1*, a negative regulator of Ras1 signaling, causes an increase in cell diameter. (d) Silencing of *Pvf1*, *2 *or *3 *individually has no effect on cell volume, but silencing of *Pvf2 *and *Pvf3 *in combination significantly reduces cell size. (e) Over-expression of *Pvf2 *or *Pvf3*, but not *Pvf1 *leads to an increase in cell volume. This affect is dependent on *Pvr*. Error bars indicate the standard error of the mean.

In order to reduce the likelihood of false positives resulting from sequence-specific off-target effects, two non-overlapping dsRNAs were used to validate each putative hit identified in the screen [33]. RNAi phenotypes for all components of the canonical Ras/MAPK signaling pathway (Figure 2a) were verified using a microscopy-based assay (Figure 2b) and by using an electronic cell counter to directly measure cell volumes (Figure 2c). This analysis revealed that dsRNAs targeting *Pvr*, *Grb2*, *Sos*, *Ras1*, *ERK *and *ksr *reduce cell size. Conversely, RNAi-induced silencing of *Gap1*, a *Ras1 *GTPase activating protein (GAP) that is a negative regulator of the pathway, led to a significant increase in cell size. However, RNAi-induced silencing of *Raf *and *MEK*, previously described as members of the Ras/MAPK signaling pathway, failed to generate equivalent changes in cell size, even when targeted using different dsRNAs (data not shown). Why this might be the case is explored below.

### *Pvf2 *and *Pvf3 *redundantly activate *Pvr *to control cell size

In order to identify the upstream signal(s) that trigger the *Pvr*-dependent increase in S2R+ cell size, we turned our attention to the previously described *Pvr *ligands Pvf1, Pvf2 and Pvf3 [24]. Since the genes for none of these three ligands were identified in the phenotypic screen (Additional data file 2), we tested for functional redundancy between the three ligands using RNAi to silence the expression of *Pvf*s in combination. Whilst silencing of individual *Pvf*s failed to induce a change in cell size, a significant reduction in cell size was observed when *Pvf2 *and *Pvf3 *were silenced together (Figure 2d), suggesting that these two ligands act redundantly to activate *Pvr*. No such synergy was seen with Pvf1 and the other ligands.

To verify this putative role for *Pvf2 *and *Pvf3 *in the control of S2R+ cell size, cells were transiently transfected with *Pvf*-containing plasmids. *Pvf *expression was then induced and cell volumes were measured using an automatic cell counter. Significantly, the expression of either *Pvf2 *or *Pvf3 *was sufficient to induce a significant increase in the average size of S2R+ cells relative to a green fluorescent protein (GFP) control (Figure 2e). By contrast, *Pvf1 *expression had no detectable effect on cell size (Figure 2e). Although it is unclear why one ligand should be non-functional in this context, previous studies have shown that different ligands operate in different settings *in vivo *[26-28,30]. Importantly, the increase in cell size induced by *Pvf2*/*3 *was observed across the population, even though transfection efficiencies remained at approximately 20%. This implies that secreted Pvf2 and Pvf3 are able to diffuse in the culture medium to trigger cell signaling in a paracrine fashion, as has been previously suggested [29]. To confirm that this effect of *Pvf*s on cell size was mediated by the Pvr receptor, an epistasis experiment was carried out in which *Pvr *RNAi cells were transfected with a construct expressing *Pvf3 *(Figure 2e), or a control plasmid. As expected, this eliminated significant differences in cell size between experimental and control populations, confirming that *Pvf*s act via *Pvr *to alter cell size.

### Pvr signaling controls cell growth

Changes in cell size can occur in the absence of alterations in the rate of cell growth via an acceleration or delay of cell division [34,35]. Such effects were clearly seen in the screen, where the silencing of *cdc25 *(*string*) caused growing cells to arrest in G2, resulting in a large increase in cell size over time (yielding a mean cell area z-score of +13.51) and a concomitant reduction in cell number. Conversely, the acceleration of cell cycle progression induced by silencing a negative regulator of the cell cycle, *wee*, reduced cell size (yielding a mean cell area z-score of -1.53). Noticeably, however, this was not accompanied by a reduction in cell number like that seen following *Pvr *or *Ras *RNAi (data not shown) [2].

**Figure 3:**
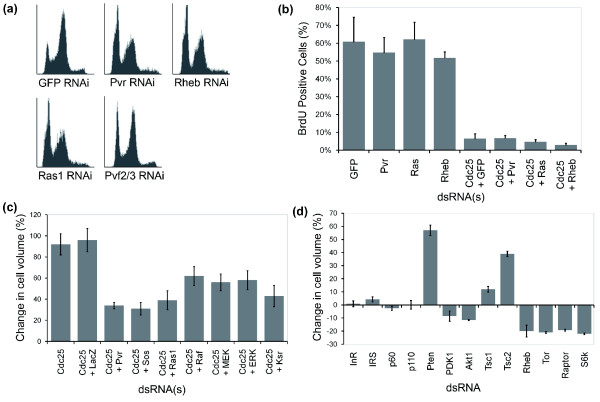
Pvf/Pvr signaling controls cell growth and G1/S progression. **(a) **FACS analysis of RNAi-treated S2R+ cells. Control cells typically exhibit a large G2 peak and a much smaller G1 peak. However, treatment with dsRNA to *Pvf2*/*3*, *Pvr*, *Ras1 *or *Rheb *causes a significant increase in the G1 peak with a concurrent decrease in the G2 peak. **(b) **BrdU labeling of cells treated with dsRNA targeting the Pvr/Ras pathway suggests that cells are still cycling. Parallel silencing of Cdc25 expression blocks cell cycle progression as expected. **(c) ***Cdc25 *(*String*) RNAi causes a significant increase in cell volume. This increase is suppressed by simultaneous silencing of *Pvr *or members of the Ras/MAPK pathway. **(d) **Chart of the mean percentage change in cell volume of RNAi treated S2R+ cells relative to control (*LacZ *RNAi) cells. Upstream components of the insulin signaling pathway do not affect cell size. However, downstream components (PDK1, Akt) exhibit a small effect. Silencing of negative regulators of both insulin (*Pten*) and Tor signaling (*Tsc1*/*Tsc2*) results in a significant increase in cell size. Conversely, silencing of positive regulators of Tor signaling reduces cell size. Error bars indicate the standard error of the mean.

Because of this link between cell cycle progression and cell size, it was important to determine whether changes in cell cycle progression contribute to the effects of Pvr/Ras signaling on cell size. To do this, we used a FACS analysis to examine the cell cycle profile of cells compromised for Pvr/Ras signaling. This revealed a significant increase in the proportion of cells in G1 in cells treated with dsRNA targeting *Pvr *or *Ras *(Figure 3a). This could be the result of a delay in the progression of cells from G1 into S-phase or the arrest of a sub-population of cells at the G1/S transition. To determine which is likely to be the case, in a second experiment we used the incorporation of bromodeoxyuridine (BrdU) as a measure of the proportion of cycling cells. BrdU was added to *Pvr*, *Ras *and *Rheb *RNAi cells 3 days after dsRNA treatment. Cells were then fixed and permeabilized 24 hours later so that incorporated BrdU could be visualized (Figure 3b). In each case, the percentage of BrdU positive cells was similar to that of the GFP RNAi control (>50%). These data strongly suggest that *Pvr*/*Ras *silencing causes a shift in the relative timing of G1/S and G2/M progression, without inducing a cell cycle arrest.

We then combined dsRNA targeting *Pvr *or other components of the Ras/MAPK pathway (*Sos*, *Ras1*, *ksr*, *Raf*, *MEK *and *ERK*) with *string *dsRNA to determine whether Pvr/Ras is required for cell growth in S2R+ cells that are unable to cycle. In each case, the FACS profile revealed a large G2 peak (data not shown), and an accompanying reduction in BrdU incorporation between days 3-4 after dsRNA treatment (Figure 3b), as expected for a *string *dsRNA-induced G2/M arrest. Significantly, however, dsRNAs targeting components of the Pvr/Ras pathway caused a significant reduction in the size of *string *RNAi cells (Figure 3c), indicating that the pathway is required for cell growth in cells arrested in G2, as it is in cycling cells. Taken together, these data suggest that the Pvr/Ras pathway is rate-limiting for the growth (accumulation of mass) of S2R+ cells and, either directly or indirectly, affects the relative time cells spend in G1 and G2.

### Tor but not insulin signaling is required for growth of S2R+ cells

Since *Pvr *has not been previously reported to control cell size, we examined the role of established growth regulatory pathways in the S2R+ cell line. Previous studies have identified the protein kinase Tor as a key regulator of cell growth in a wide variety of eukaryotic systems [32]. In the canonical Tor pathway, the small GTPase Rheb activates the Tor/Raptor complex, which phosphorylates ribosomal S6-kinase to stimulate cell growth [32]. However, our genome-wide RNAi screen only identified a single member of the Tor pathway, *Rheb*, as a putative regulator of cell size. A closer examination of the screen data revealed that our failure to identify other components of the Tor signaling pathway was due in part to the stringent cut-off employed in the computational analysis to reduce the number of false positives. In fact, *Tor*, *Raptor *and *S6k *silencing was associated with a small, but measurable decrease in cell area (z less than -1.6 in each case), suggesting that the Tor pathway does indeed play a role in the control of cell growth in S2R+ cells. To confirm this, we generated non-overlapping dsRNAs for each pathway component and directly measured cell sizes using an electronic cell analyzer. All core members of the canonical Tor pathway displayed the expected RNAi phenotype. Silencing positive regulators of the pathway (*Rheb*, *Tor*, *Raptor *and *S6k*) led to a significant decrease in cell size (Figure 3d). Conversely, dsRNAs targeting either of the two negative regulators of the pathway, *Tsc1 *and *Tsc2 *(which together form a *Rheb *GAP), increased cell size (Figure 3d). Furthermore, FACS analysis revealed that *Rheb *or *Tor *RNAi leads to an increase in the proportion of cells in G1, similar to that seen in *Pvr *RNAi cells (Figure 3a). These results confirm that the canonical Tor pathway controls cell growth in S2R+ cells, as previously demonstrated in S2 cells [36,37] and *in vivo *[10].

Tor has been shown to act downstream of insulin-induced receptor tyrosine kinase (RTK) signaling to control *Drosophila *cell growth *in vivo *[10]. Moreover, insulin has been shown to stimulate the growth of *Drosophila *cells *in vivo *[38] and in fly cell culture [39]. This signal is mediated by the insulin receptor (*InR*). The activated receptor recruits the insulin receptor substrate (*IRS*) adaptor protein, which binds the regulatory (*p60*) subunit of class I PI3K, enabling the catalytic (*p110*) subunit to convert the phospholipid PIP2 to PIP3 in the membrane. PIP3 then recruits several downstream targets, most notably PDK1, to the membrane, to induce the phosphorylation and activation of Akt/PKB, which goes on to inactivate Tsc1/2 to stimulate the Tor pathway. In analyzing the role of insulin signaling in the growth of S2R+ cells, we first verified that insulin is able to alter their growth. As expected for a cell line with an intact insulin signaling pathway, the addition of insulin to the medium of these cells increased the rate of proliferation and average cell volume (data not shown). This does not mean, however, that insulin signaling is required for normal S2R+ cell growth. To test whether or not this was the case, we measured cell size following RNAi-induced silencing of pathway components. Knocking down of the upstream components *InR*, *IRS*, *p60 *or *p110 *had no effect on cell size (Figure 3d), even though this was sufficient to fully (*InR*, *IRS*, *p60*) or partially (*p110*) [40] attenuate the insulin-induced phospho-Akt response (data not shown), while *PDK1 *or *Akt *silencing induced a small reduction in cell size (Figure 3d). These experiments suggest that while the insulin pathway is operational in S2R+ cells, it is not rate-limiting for size control in this cell line under normal cell culture conditions.

### The PI3K and MAPK pathways act in parallel to relay the *Pvr *growth signal

Having established important roles for *Pvr *and *Ras1 *in the regulation of S2R+ cell growth, an RNAi epistasis analysis was used in an attempt to delineate downstream signaling events in more detail, and to better understand the reason for the minor phenotypic consequences of using RNAi to deplete several well-established Ras targets. To begin this analysis we used negative regulators of cell growth signaling in this system, *Gap1 *and *Tsc2*, as genetic landmarks to position positive regulators within the pathway.

**Figure 4:**
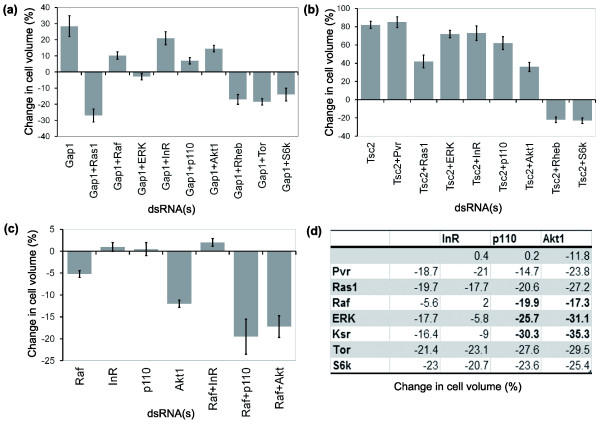
Pvf/Pvr signaling activates a network of signaling modules upstream of the Tor pathway. **(a) ***Gap1 *RNAi epistasis. *Ras1*, *Rheb*, *Tor *and *S6k *dominate in their effect on cell size, *ERK *suppresses the *Gap1 *RNAi phenotype, whereas *Raf*, *p110 *and *Akt *only partially ameliorate the large increase in cell size seen following *Gap1 *RNAi. The insulin receptor has little effect in this assay. **(b) ***Tsc2 *RNAi epistasis. *Rheb *and *S6k *dominate, placing them genetically downstream of *Tsc2*. *Pvr*, *Ras1 *and members of the MAPK and PI3K pathways fail to have a dramatic impact on the *Tsc2 *RNAi phenotype. **(c) ***Raf *(*pole hole*) RNAi epistasis. Silencing of *Raf *leads to a minor reduction in cell size. However, silencing of *Raf *in conjuction with *p110 *or *Akt1 *causes a large reduction in cell size, like that seen in *Pvr*, *Ras*, *Tor*, *Rheb*, and *S6K *RNAi experiments. **(d) **MAPK and PI3K pathway genetic interactions. *Raf*, *ERK *and *Ksr *all show additive or synergistic genetic interactions with *p110 *and *Akt1*, but not with *InR*. Furthermore, these genetic interactions are stronger than those seen when combining *p110 *or *Akt1 *dsRNA with dsRNA targeting upstream or downstream pathway components of this putative growth signaling network (*Pvr*/*Ras1 *and *Tor*/*S6k*, respectively). Error bars indicate the standard error of the mean.

We began by using RNAi to modify the Gap1 phenotype. As expected, the *Gap1 *RNAi-induced increase in cell size could be suppressed by *ERK *RNAi, and reversed by dsRNA-mediated silencing of *Ras1*, and by reductions in the expression of downstream components of the Tor signaling pathway, Rheb, Tor and S6K (Figure 4a). Interestingly, however, direct targets of Ras1, Raf and p110 only partially suppressed the effects of *Gap1 *RNAi (Figure 4a), mirroring the results of single *Raf *and *p110 *RNAi experiments (Figures 2c and 3c). These results suggest that both the MAPK and PI3K pathways contribute to the communication of the growth signal downstream of Ras1.

We then repeated this epistasis analysis in a background in which the gene for the Rheb GAP Tsc2 was silenced, deregulating Rheb activity to increase cell growth (Figure 3c). Once again, although several dsRNAs (*Ras1*, *p110 *or *Akt*) reduced the extent of the cell size increase seen following *Tsc2 *RNAi, the Tsc2 phenotype dominated in each case (Figure 4b), placing these genes genetically upstream of *Tsc2*. Although different pathway members (for example, Pvr versus Ras1) exhibited minor differences in their ability to suppress the Tsc2 phenotype, we believe that this is likely to reflect the fact that epistasis experiments are inherently sensitive to gene-specific differences in the kinetics of RNAi knockdown. Only Rheb and S6k strongly attenuated the Tsc2 phenotype, implying that they function downstream of Tsc2, as previously reported [32]. Taken together (compare Figure 4a and 4b), these results suggest that Pvr/Ras signaling is likely to operate upstream of the Tor pathway in controlling cell growth in S2R+ cells, although we cannot exclude the possibility that Ras and Tor signaling operate in parallel.

These results focused our attention on the function of intermediate pathway components that have a minor impact on cell growth when targeted using RNAi (Figures 2c and 3d). Since Ras has been shown to signal directly to both PI3K and Raf in other systems [41], we decided to use combinatorial RNAi experiments to test whether p110 and Akt might cooperate with Raf in relaying the growth signal downstream of Ras1 in S2R+ cells (Figure 4c). This analysis revealed a set of additive and synergistic interactions between components of the MAPK and PI3K pathways (Figure 4c, d). This was clearest for *p110*, since the reduction in cell size observed following silencing of *p110 *together with either *Raf*, *ksr *or *ERK *was equal to or greater than the sum of phenotypes observed in RNAi experiments targeting these genes independently (Figure 4c, d and data not shown). In addition, there was an additive effect of targeting Akt and these components of the MAPK pathway. Since *InR *RNAi failed to enhance the effect of *Raf *silencing (Figure 4c), this synergy between *Raf *and *p110*/*Akt *is unlikely to be the result of a parallel input from insulin signaling. Instead, because *p110*/*Akt *RNAi did not synergize with *Pvr *and *Ras1 *RNAi (Figure 4d), the PI3K pathway likely functions downstream of Ras1 in this growth assay, as has been described in other systems [41]. Taken together, these results suggest that signals relayed by both the MAPK and PI3K pathways cooperate in growth signaling. Indeed, both Akt and ERK have been shown to phosphorylate and inactivate Tsc2 in mammalian systems [42-45]. Thus, the Tsc1/Tsc2 complex may serve as a hub to integrate growth signals.

### *Pvr *controls cell size in other *Drosophila *cell lines and in larval hemocytes

**Figure 5:**
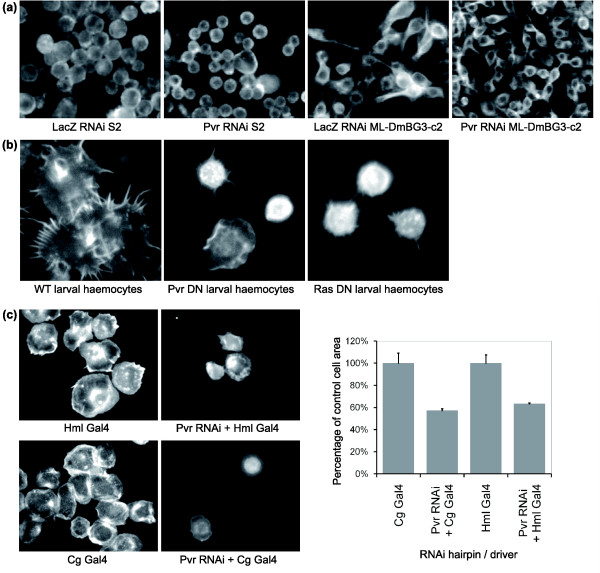
*Pvr *controls cell growth in a variety of cell lines and in *Drosophila *hemocytes. **(a) **F-actin staining of *Drosophila *S2 and ML-DmBG3-c2 (neuronal) cells treated with dsRNA to *LacZ *or *Pvr *reveals a reduction in cell size in *Pvr *RNAi cells relative to control. **(b) ***Pvr *and *Ras1 *dominant negative constructs were expressed in larval hemocytes *in vivo *using the Cg-Gal4 driver. Hemocytes were extracted, allowed to adhere to conconavalin A and then fixed and stained for F-actin. Cell area was measured from images of hemocytes (see Materials and methods). Both *Pvr *and *Ras1 *DN constructs caused a significant reduction in hemocyte cell area relative to wild-type (WT). **(c) **Driving expression of *Pvr *RNAi constructs in hemocytes using either Cg-Gal4 or Hml-Gal4 causes a significant decrease in cell size relative to control (driver or hairpin only) cells. Cells were stained for F-actin. Control and experimental images in (b, c) were taken at the same magnification. Error bars indicate the standard error of the mean.

Having identified a Pvr signaling pathway that is rate-limiting for the growth of the S2R+ hemocyte-derived *Drosophila *cell line, we extended this analysis to investigate possible implications for the growth of other cells. First, we examined the effects of *Pvr *silencing in a variety of other *Drosophila *cell lines. *Pvr *showed a strong cell size phenotype in both the S2 hemocyte cell line and the neuronal cell line ML-DmBG3-c2 (Figure 5a), implying that Pvf/Pvr autocrine signaling is a common feature of the growth of *Drosophila *cell lines in culture.

To test whether *Pvr *might play a similar role in the regulation of cell size *in vivo*, we extracted hemocytes from *Drosophila *larvae containing *Pvr *or *Ras *RNAi constructs under the control of two different hemocyte drivers (Hml-Gal4 and Cg-Gal4). In both cases, we used upstream activation sequence (UAS)-GFP as a marker to confirm that transgenes were being expressed in these primary cells. In order to estimate cell size, mean cell area was measured after GFP-labeled cells had been given time to spread on an adhesive concanavilin A-coated surface. As controls, we also measured the spread area of cells lacking either the driver (data not shown) or the RNAi hairpin. In this experiment, *Pvr *silencing or the expression of a dominant negative *Pvr *or *Ras *construct led to a significant reduction in the size and number of hemocytes relative to control experiments (Figure 5b and data not shown). Although *Ras1 *over-expression has previously been shown to cause an increase in larval hemocyte number [46], which necessarily requires coincident cell growth and division, these data suggest that Ras/MAPK signaling also plays a role in mass accumulation. Thus, *Pvr *and *Ras1 *control the growth, proliferation [29] and viability [22] of *Drosophila *hemocytes *in vivo*.

## Discussion

In this study, we have used an automated image analysis pipeline to screen through images from a high-content, genome-wide RNAi screen for genes whose activity is rate-limiting for the growth of *Drosophila *cells in culture. In doing so, we identified a number of known and novel genes regulating cell size. Interestingly, this screen identified a novel role for autocrine signaling through Pvfs and the receptor tyrosine kinase Pvr in the control of the autonomous growth of *Drosophila *cells in culture. Previous studies have suggested roles for Pvf/Pvr signaling in the control of cell migration [23,27,30], morphogenesis [25,26,31], cell viability [22] and proliferation [28,29]. However, to our knowledge this is the first clear example of this pathway controlling cell size. This reduction in the size of *Pvr *RNAi cells was accompanied by a reduction in cell proliferation, as revealed by reduced cell numbers in the absence of significant apoptosis (data not shown), and by a delay in the passage of cells through G1 and into S phase.

One interpretation for these observations is that *Pvr *knockdown might trigger autophagy in S2R+ cells. Indeed, one of the first responses that cells exhibit when confronted with unfavorable growth conditions is to shrink in size and trigger autophagy to meet their energy demand during poor nutritional conditions. It is also possible that a loss of cell mass following *Pvr*/*Ras *RNAi contributes to the small cell phenotype, should this growth signaling pathway regulate both growth and autophagy in S2R+ cells.

Next, we used an RNAi epistasis analysis to delineate this Pvr growth-signaling pathway. Interestingly, whilst the effects of dsRNAs targeting upstream components (*Pvr*, *Sos *and *Ras*) of the Pvr/Ras pathway were much greater than the effects of targeting individual downstream components (*Raf*, *Ksr *and *ERK*), dramatic reductions in cell size were observed when dsRNAs were combined that target both PI3K and MAPK arms of the downstream signaling pathway (Figure 4d). These data suggest that the growth signal downstream of Ras requires the combined activity of downstream PI3K and MAPK signaling modules. Our data also suggest that these signaling pathways act upstream of the Tor pathway in S2R+ cells, as shown in other systems [42-45]. Although this type of growth signal integration has not been previously reported in *Drosophila*, *Ras1 *has been shown to influence both growth and G1/S cell cycle progression *in vivo *[47] and *Ras1 *has been shown to crosstalk to *dp110 *in the control of *in vivo *cell and tissue growth [6].

In the context of development, crosstalk between signaling pathways, like that seen in our analysis, could help to integrate information from different types of intrinsic and extrinsic cues in order to aid cellular decision making. Alternatively, as seen in this study, the use of parallel signaling modules (PI3K and MAPK in this case) may serve to buffer cellular behavior from changes in the relative levels of different input signals. However, *in vivo*, we would not expect to observe many instances of autocrine growth signaling, since this is inherently hard to regulate. Indeed, *in situ *hybridization studies in *Drosophila *embryos suggest that receptors such as Pvr are expressed in specific populations of cells, such as hemocytes, that do not express any of the corresponding ligands Pvf1-3 [23]. Similarly, Pvf secretion is restricted to particular compartments in pupal stages, and when deregulated can cause tumorous growth [29]. These data suggest that mutations leading to *Pvr *and *Pvfs *co-expression may contribute to the establishment of autonomous cell growth during the establishment of *Drosophila *cell lines in media based on bovine serum, which lacks strong activators of ERK and PI3K signaling [40]. In the future it will therefore be interesting to investigate the mechanisms used to ensure that the *in vivo *expression of ligand receptor pairs, like Pvfs and Pvr, remains mutually exclusive, and to reveal how these controls are deregulated during the establishment of a *Drosophila *cell line. We would expect this information to be useful in the establishment of new cell lines and in furthering our understanding of the processes leading to the deregulated expression of ligand-receptor pairs during the development of a variety of cancers [48,49].

## Conclusion

This study presents evidence for a novel role for autocrine Pvf/Pvr signaling in cell growth, both in cell culture and *in vivo*. The *Drosophila *homologue of the mammalian PDGF/VEGF receptor family acts upstream of Ras, and diverging MAPK and PI3K signaling modules. Since *Pvf2*/*3 *and *Pvr *show mutually exclusive patterns of expression *in vivo*, these data suggest that co-expression of this receptor-ligand pair plays a key role in driving cell autonomous growth during the establishment of *Drosophila *cell lines, as has been suggested to occur during tumor development.

## Materials and methods

### *Drosophila *cell culture

S2R+ cells were grown in Schneider's medium (Invitrogen, Carlsbad, California, USA) or Shields and Sang M3 insect medium (Sigma-Aldrich, St Louis, Missouri, USA) with 10% heat-inactivated fetal bovine serum (Sigma-Aldrich, St Louis, Missouri, USA) and penicillin-streptomycin (Sigma-Aldrich). S2 cells were grown in InsectExpress media with L-Glutamine (PAA Laboratories, Pasching, Austria). ML-DmBG3-c2 cells were cultured in M3 media supplemented with fetal bovine serum, antibiotics and 10 μg/ml bovine insulin (Sigma-Aldrich, St Louis, Missouri, USA). *Drosophila *S2R+ cells were transiently transfected using the CellFectin (Invitrogen, Carlsbad, California, USA) lipid transfection reagent according to the manufacturer's protocol. Where necessary, gene expression was induced by addition of 1 mM CuSO_4 _solution.

### RNAi

dsRNA templates were amplified by PCR from genomic DNA using pairs of gene-specific primers. dsRNA synthesis was performed using the T7 Megascript kit (Applied Biosystems, Foster City, California, USA). RNA preparations were purified using PCR96 cleanup plates (Millipore, Billerica, Massachusetts, USA) attached to a vacuum pump. Purified RNAs were resuspended in Tris-EDTA buffer (TE) and annealed by heating at 65°C for 10 minutes and cooling slowly. Typically, cells suspended in serum-free medium were mixed with dsRNA to give a final concentration of 30 μg/ml then plated into tissue culture dishes and incubated at 24°C for 30 minutes. Subsequently, three volumes of complete medium was added and cells were grown for 5-7 days at 24°C to allow for protein turnover [50].

### Cytoskeletal staining and image acquisition

Cells in 384-well plates were washed with phosphate buffered saline (PBS) and fixed for 10 minutes in 4% formaldehyde (Polyscience, Niles, Illinois, USA). After fixation cells were permeablized by washing with PBS containing 0.1% Triton-X-100 (PBS-T), then blocked with 5% bovine serum albumin (Sigma) in PBS-T for 20 minutes. For staining, cells were first incubated with 1:500 α-Tubulin antibody (Sigma) in PBS-T containing 1% bovine serum albumin overnight at 4°C. Cells were then washed twice with PBS-T and incubated with fluoro-isothiocyanate (FITC) anti-mouse IgG secondary antibody (The Jackson Laboratory, Bar Harbor, Maine, USA) combined with TRITC-Phalloidin (Sigma) and DAPI (Sigma) for 2 hours. For BrdU experiments, BrdU was added to the culture medium 3 days after the addition of RNAi. Cells were then fixed, acid washed and stained 24 hours later, using TRITC-labeled anti-BrdU antibodies to reveal the extent of BrdU incorporation into DNA. In each case, fluorescent images were acquired using an automated Nikon TE2000E microscope with a 20× objective and HTS (high throughput screening) MetaMorph software (Molecular Devices, Sunnyvale, California, USA) running an automated stage and shutter (Prior, Cambridge, UK), and a Roper CoolSNAP cooled-coupled device camera.

### Computational image analysis and score normalization

Image analysis was performed using the image analysis toolkit in Matlab (Mathworks, Natick, Massachusett, USA). F-actin and microtubule stained images were processed to remove cell clumps and background, leaving the cell monolayer. DNA stained images were segmented to identify nuclei in the cell monolayer. Mean cell area was calculated as the area of the monolayer divided by the number of nuclei. Raw scores from image analysis were normalized to correct for systematic differences between assay plates. Normalization was performed using the CellHTS package [19], part of the Bioconductor suite of biological data analysis packages for the R statistical computing environment. Briefly, mean cell area scores were normalized using median centering per plate, and screen z-scores were calculated using the screen median and the median absolute deviation (MAD). Replicate scores from different image sites in the same well were summarized using the closest to zero function (equivalent to taking the minimum, independent of sign) to calculate a single z-score for each screen well. The proportion of nuclei that had undergone division was established by computational image analysis of BrdU and DAPI images.

### Cell size and cell cycle measurements

Cell volume was measured using a CASY cell counter and analysis system (Scharfe System, Reutlingen, Germany). Cells diluted 1:101 in CasyTon reagent (Scharfe System) were measured in triplicate. The mean cell volume for each treatment was calculated as the average peak volume from three independent readings. For each experiment the peak cell volume (the peak in the histogram of individual cell volumes) for at least ten control wells measured in triplicate was used to establish a solid baseline for comparison. Since control cell size varied between experiments, it was necessary to normalize scores for each experiment before summarization. Thus, volumes were converted to the percentage of mean control cell volume. Percentage of mean control cell volume from at least two independent experiments were averaged and used to construct bar charts. For cell cycle profiles, the cells were fixed in 70% ethanol at -20°C and subsequently resuspended in PBS containing 50 μg/ml propidium iodide and 60 μg/ml RNaseA. The profiles were acquired on a FACSanto anlyzer, using FACSDiva software (Becton Dickinson, Franklin Lakes, New Jersey, USA). All cells were in log growth phase during the course of the experiments.

### *In vivo *methods

Cg-Gal4, Hml-Gal4, UAS-GFP and UAS-RasDN lines were obtained from the Bloomington stock centre. The UAS-PvrDN line was a gift from P Rorth and *Pvr *RNAi lines were gifts from Benny Shilo. Late third instar larvae were washed and the integument was disrupted in the latero-posterior region without organ disruption. The circulating hemocytes were directly collected in M3 medium. In each case, pooled hemocytes from several larvae were plated on conconavalin A coated 384-well plates and allowed to spread flat on this substrate for 2 hours. Attached hemocytes were fixed, stained and imaged as above and cell area was measured computationally. UAS-GFP was used to confirm Gal4 expression in larval hemocytes.

## Abbreviations

BrdU: bromodeoxyuridine; dsRNA: double-stranded RNA; FACS: fluorescence activated cell sorting; GAP: GTPase activating protein; GFP: green fluorescent protein; MAPK: mitogen-activated protein kinase; PBS: phosphate-buffered saline; PDGF: platelet-derived growth factor; PI3K: phospho-inositide-3-kinase; RNAi: RNA interference; UAS: upstream activation sequence; VEGF: vascular endothelial growth factor.

## Authors' contributions

DS performed the computational RNAi screen analysis and the subsequent hit validation and epistasis analysis. PD performed a visual analysis of the RNAi screen and the over-expression and FACS studies. DS and BB carried out the *in vivo *analysis. DS and BB designed the experiments and wrote the manuscript. All authors read and approved the final manuscript.

## Additional data files

The following additional data are available with the online version of this paper. Additional data file 1 describes in full the top cell size hits from computational analysis of images from a high-content genome-wide RNAi screen. Additional data file 2 provides details of computational image analysis scores for all MAPK, Tor and insulin pathway components.

## Supplementary Material

Additional data file 1 This list was generated using a mean cell area z-score threshold of less than -2. dsRNAs giving rise to visually confirmed phenotypes are highlighted in yellow. Columns J-L: 'Y' indicates a hit, 'N' indicates that a dsRNA was screened but not hit and '-' indicates that no dsRNA targeting the gene in question was screened. Click here for file

Additional data file 2 Columns I-K: 'Y' indicates a hit, 'N' indicates that a dsRNA was screened but not hit and '-' indicates that no dsRNA targeting the gene in question was screened. All image analysis scores along with original images and dsRNA primer and amplicon details from the entire screen are available online in the FLIGHT database [20]. Click here for file
